# Supplementary material for: Optimizing mechanical ventilation: Personalizing mechanical power to reduce ICU mortality ‐ a retrospective cohort study
Source: PLoS One. 2025 Feb 13;20(2):e0318018. doi: 10.1371/journal.pone.0318018 (PMC11825045; doi:10.1371/journal.pone.0318018)
Supplement: S1 Table — (DOCX) [file pone.0318018.s001.docx]

**Supplementary table 1: Multivariate Stepwise Backward Analysis**

| **Variables** | | **B** | **S.E.** | **Wald** | **df** | **Sig.** | **Exp(B)** | **95% C.I.for EXP(B)** | |
| --- | --- | --- | --- | --- | --- | --- | --- | --- | --- |
|  |  |  |  |  |  |  |  | **Lower** | **Upper** |
| **48 hours TWA-MP** |  | .011 | .007 | 2.734 | 1 | .098 | 1.011 | .998 | 1.024 |
| **Age** | **18-39** |  |  | 79.501 | 5 | .000 |  |  |  |
|  | **40-49** | .121 | .317 | .145 | 1 | .704 | 1.128 | .606 | 2.100 |
|  | **50-59** | .533 | .279 | 3.645 | 1 | .056 | 1.704 | .986 | 2.946 |
|  | **60-69** | .941 | .264 | 12.700 | 1 | .000 | 2.562 | 1.527 | 4.299 |
|  | **70-79** | 1.451 | .260 | 31.039 | 1 | .000 | 4.266 | 2.561 | 7.107 |
|  | **> 80** | 1.768 | .286 | 38.237 | 1 | .000 | 5.860 | 3.346 | 10.264 |
| **Highest Lactate** |  | .153 | .024 | 39.392 | 1 | .000 | 1.165 | 1.111 | 1.222 |
| **Highest pH** |  | -2.441 | 1.396 | 3.057 | 1 | .080 | .087 | .006 | 1.344 |
| **Lowest APTT** |  | .019 | .007 | 7.552 | 1 | .006 | 1.020 | 1.006 | 1.034 |
| **Lowest BE** |  | .022 | .013 | 2.921 | 1 | .087 | 1.022 | .997 | 1.048 |
| **Lowest Pco2** |  | -.027 | .016 | 2.826 | 1 | .093 | .973 | .943 | 1.005 |
| **Lowest pH** |  | -2.375 | 1.411 | 2.834 | 1 | .092 | .093 | .006 | 1.477 |
| **Constant** |  | 32.703 | 12.976 | 6.352 | 1 | .012 | 159410063812341.250 |  |  |
