# Supplementary material for: Optimizing mechanical ventilation: Personalizing mechanical power to reduce ICU mortality ‐ a retrospective cohort study
Source: PLoS One. 2025 Feb 13;20(2):e0318018. doi: 10.1371/journal.pone.0318018 (PMC11825045; doi:10.1371/journal.pone.0318018)
Supplement: Attachment — Submitted filename: Response to Reviewers.docx [file pone.0318018.s002.docx]

**Response to Reviewers**

We would like to express our sincere gratitude to the reviewers for their valuable feedback and insightful comments on our manuscript. Your detailed review and constructive suggestions have been instrumental in improving the quality of our work. We have carefully considered each comment and have made the necessary revisions as outlined in our detailed responses below.

**Reviewer #1:** had no comments

**Reviewer #2:**

**1. Please provide more details on the machine learning models, including feature selection process and hyperparameter tuning.**
Details about the model has been added (Line 137-148). **2. Please expand on the proposed individualization method for mechanical ventilation settings. How exactly would this be implemented clinically?**
More detailed explanation was added (Line 150-166) **3. The authors should include a multivariable analysis to assess the independent association of mechanical power with mortality after adjusting for confounders.**
Added as a supplementary table (S1 Table) **4. Please consider external validation of the findings using another ICU dataset, if possible.**
Thank you for your suggestion. We agree that external validation using another ICU dataset would enhance the robustness of our findings. However, due to current data access constraints, conducting external validation is not feasible within the scope of this study. We have thoroughly validated our findings internally and have highlighted the need for external validation in future research as a limitation in the revised manuscript. **5. The authors should clarify how the "safe upper limits" for mechanical power were determined statistically.**
Details added at line 127-132 **6. The counter-intuitive findings in severely hypoxemic patients warrant further discussion and analysis.**
Details added at line 253-255 **7. More context on how these findings compare to existing literature on mechanical power thresholds would be needed.**
Details added at line 262-264 **8. The clinical implications and potential next steps for research should be elaborated on in the discussion.**

Details added at line 285-295

**Reviewer #3:**

**1. Clarity in Background and Literature Context: The background section is concise and informative on the research but lacks depth in contextualizing existing literature. It gives the impression that the field of mechanical ventilation (including MP) and it’s relation to ICU mortality is less explored by machine learning and deep learning that it actually is. I would recommend expanding the background to include a more comprehensive review.**

Thank you for your thoughtful feedback. We appreciate your suggestion to expand the background. However, we have deliberately kept this section concise to maintain the focus of the manuscript on the study's objectives and avoid redundancy with existing comprehensive reviews in the field. The current background aims to provide sufficient context to support the study rationale, focusing on the most relevant studies directly related to our work. We believe that the current level of detail adequately sets the stage for our research without overextending into a broader literature review.

**2. Wrong primary outcome: The research uses the ICU mortality as primary outcome, and this might not be the best outcome for this type of study. ICU mortality relies on several factors not related to mechanical ventilation, and patients with long stays at the ICU usually not die because of bad mechanical ventilation. Usually, other outcomes are used in this type of studies because the represent better a bad ventilation, like ventilator free days, time to successful extubation, or short or medium term mortality.**

Thank you for your valuable input regarding the choice of the primary outcome. We acknowledge that ICU mortality is influenced by multiple factors beyond mechanical ventilation. However, it remains a critical outcome of clinical significance that aligns with our study’s objective to explore overall patient prognosis in the ICU setting. We have carefully considered various outcomes and have chosen ICU mortality to provide a broad evaluation of the impact of mechanical ventilation within the ICU environment. Additional outcomes, such as ventilator-free days, are secondary considerations but are beyond the primary scope of this study

**3. Data transparency: The journal inquires in the guidelines about the importance on data sharing, while it is understandable that the data does not belong to the researchers, it would be ideal that the researchers share the code to extract the data from the original database.**

We fully agree with the need to share resources that enhance the reproducibility of our research. The codes used to extract the data are already available, and we have added further details about this in the methodology section of the manuscript. These codes are accessible in the repository we have referenced, ensuring that other researchers

can replicate our data extraction process within the constraints of data access permissions.

**4. Methodological transparency. The methodology of the manuscript lacks detail in data processing, cleaning, missing filling and division in datasets. All of this information is needed to be able to reproduce the research. The subsection “Mortality prediction” of the manuscript it’s the only section in which a bit of the methodology of the data pipeline is explained and it clearly lacks detail. My recommendation would be to write one subsection dedicated to the data pipeline, and separate it from the methodology of the different data experiments.**

Thank you for the comment, Further details were added in the methodology section.

**5. Conclusion not supported by the results. The results in the survival analysis and figure 3 show that there is NO statical significance on the survival probability based on the upper limits identified. The researchers are mistaken and the conclusion based on this result is wrong.**

Thank you for your comment. We would like to clarify that our conclusion does not imply statistical significance in the survival probability based on the identified upper limits. Rather, our conclusion focuses on the observed relationship between mechanical power and ICU mortality and highlights the promising potential of machine learning models in predicting outcomes. The intent is to emphasize the trend observed and the innovative application of ML models in this context, rather than overstate the findings as statistically significant. We believe this perspective adds value to the ongoing discussion about personalized and data-driven approaches in managing mechanically ventilated patients.

**Minor issues:
1. Definition of inadequate data. In abstract and methodology (line number 87) the authors write that they eliminated patients with inadequate data. The manuscript should be more concrete on what is inadequate data and how the authors defined it and processed it.**

Further details have been added in line 91 – 98  **2. Data extraction. The authors talk about data extraction only at line 93, but code is unavailable and there is almost not real description on how data was extracted (procedure, technical details, tools used). To be able to reproduce the research, this is needed.**

Further details have been added in line 100-110

**3. Statistical and Machine Learning analysis. The authors state at the lines 124-126 that they developed several models. Sadly, they only expose the results of XGBoost. Results of all models should be shown in results to contextualize the good results from XGBoost.**

Further details have been added in Table 5

**4. Relation between identified MP limits and individualization methods. While the individualization methods for mechanical ventilation settings are a very interesting approach, the authors don’t seem to connect the identified MP limits with the individualization methods. It would be appropriate if they could relate both parts of the paper, if not, is like two different investigations separated and they might want to focus on one for the paper.**

Further details have been added in line 137-166

**5. Result of general limit for MP is not explained on the methodology. At line 167, the authors state that an upper limit of 16.51 J/min was determined for all patients, nonetheless, the authors don’t mention on the methodology how they determine the limit.**

Further details have been added in line 127-132

**6. Provide more details on the algorithm or function used for optimizing MV settings based on MP and other physiological variables. In the subsection of the methodology “Individualization of MV settings”, the authors explain the process of individualization of MV settings, but they don’t explicitly say how the new values of MV are calculated. Ar they random approximation? A inequation being solved? Suggested by any algorithm? They should explain more this part.**

Further details have been added in line 150-166

**7. More in-depth discussion. During the discussion, the authors mainly repeat the results obtained. While I understand that they want to reinstate their results, they should focus more on the clinical meaning of this results.**

Fine tuning in the discussion part was done, Thank you for your insight

**8. More in-depth limitations. More limitations of this study should be mentioned like that the research is done only on PCV patients, the use ICU mortality outcome (or not if changed to sorth/medium days ICU mortality), and potential biases in the individualization method’s predictions.**

Limitations have been adjusted

**9. Table 1. This table has feature “Admitting speciality” with over 10 categories. Some of these categories have less than 10 patients between both groups. As a data cleaning process this feature should be grouped into main categories and “other” category.**

Results were cleaned further

**10. Table 2. Table has laboratory data, but the extraction is not mentioned in methodology. It should be added to the methodology if this is important.**

Details have been added in the methodology, thank you for the insights
