# Supplementary material for: Optimizing mechanical ventilation: Personalizing mechanical power to reduce ICU mortality ‐ a retrospective cohort study
Source: PLoS One. 2025 Feb 13;20(2):e0318018. doi: 10.1371/journal.pone.0318018 (PMC11825045; doi:10.1371/journal.pone.0318018)
Supplement: Attachment — Submitted filename: Response to Reviewers V2.docx [file pone.0318018.s003.docx]

**Response to Reviewers**

We would like to express our sincere gratitude to the reviewers for their valuable feedback and insightful comments on our manuscript. Your detailed review and constructive suggestions have been instrumental in improving the quality of our work. We have carefully considered each comment and have made the necessary revisions as outlined in our detailed responses below.

**Reviewer #3:** had no comments

**Reviewer #2:**

**1. Machine Learning Models: While the authors have added more details about the models (lines 137-148), further information on the feature selection process and hyperparameter tuning would enhance reproducibility.**

Edis have been added in line 152-154 **2. Individualization Method: The expanded explanation (lines 150-166) improves understanding, but more concrete examples of how this method would be implemented clinically would be beneficial.**

Edit have been added in line 175-180 **3. Multivariable Analysis: The addition of the supplementary table (S1 Table) addressing the independent association of mechanical power with mortality is valuable. This should be referenced and discussed in the main text.**

Edit have been added in line 203-210 **4. External Validation: While understandable due to data access constraints, the lack of external validation remains a limitation. The authors should discuss potential implications of this limitation more thoroughly.**

Edit have been added in line 347 - 352 **5. Safe Upper Limits: The added details (lines 127-132) clarify the statistical determination of safe upper limits. However, a brief discussion on the clinical significance of these thresholds would be helpful.**

Edits have been added at line 132 – 138 **6. Severely Hypoxemic Patients: The additional discussion (lines 253-255) on the counter-intuitive findings in severely hypoxemic patients is appreciated, but further exploration of potential mechanisms or implications would strengthen the paper.**

Edit have been added in line 274-281 **7. Comparison to Existing Literature: The added context (lines 262-264) comparing findings to existing literature is helpful. Expanding this comparison could provide readers with a better understanding of how this study advances the field.**

Edit have been added in line 291 - 302 **8. Clinical Implications: The elaboration on clinical implications and future research directions (lines 285-295) improves the paper's impact. Consider discussing potential challenges in implementing the proposed individualized approach in clinical practice.**

Edit have been added in line 325-331
