# Supplementary material for: Optimizing mechanical ventilation: Personalizing mechanical power to reduce ICU mortality ‐ a retrospective cohort study
Source: PLoS One. 2025 Feb 13;20(2):e0318018. doi: 10.1371/journal.pone.0318018 (PMC11825045; doi:10.1371/journal.pone.0318018)
Supplement: Attachment — Submitted filename: Response to Reviewers V3.docx [file pone.0318018.s004.docx]

**Response to Reviewers**

We would like to express our sincere gratitude to the reviewers for their valuable feedback and insightful comments on our manuscript.

**Reviewer #3:** had no comments

**Reviewer #2:**

1. Statistical Analysis:
- Please address the apparent discrepancy between univariate and multivariate analyses regarding MP's association with mortality. The discussion in lines 203-210 needs expansion.

**Further elaboration was added. line ( 206-212 )**

- The Cox survival analysis results (p=0.35) need clearer interpretation, particularly given its implications for your safe limits hypothesis

**Further elaboration has been added (lines 232–233). Additionally, the discussion already provides an explanation of this result.**

- Consider additional sensitivity analyses to validate your identified MP thresholds
**Thank you for your comment. This study is intended as a proof of concept, and the authors believe the current detailed analysis sufficiently demonstrates the validity of the findings.**

2. Machine Learning:
- Provide more details about the model development process, particularly:
**-Feature selection criteria beyond correlation thresholds: Added (lines 149–152).**

**-Rationale for choosing XGBoost over Stacking despite lower performance: Added (lines 245–248).**

3. Discussion:
- Strengthen the comparison with existing literature

**We appreciate the importance of situating our findings within the broader context of prior research. In the revised manuscript, we believe that the current discussion already provides sufficient comparisons to key studies as referenced**

- Elaborate on the unexpected findings in severe hypoxemia patients

**Thank you for the comment. The authors believes that the explanation in (line 278-293) is sufficient.**

- Add future research directions

**Added in line ( 373-378)**
